# Editorial: Origins of the Resting-State fMRI Signal

**DOI:** 10.3389/fnins.2020.594990

**Published:** 2020-10-27

**Authors:** J. Jean Chen, Peter Herman, Shella Keilholz, Garth J. Thompson

**Affiliations:** ^1^Rotman Research Institute, Baycrest Health Sciences, Toronto, ON, Canada; ^2^Department of Medical Biophysics, University of Toronto, Toronto, ON, Canada; ^3^Department of Radiology and Biomedical Imaging, Yale University, New Haven, CT, United States; ^4^Wallace H. Coulter Department of Biomedical Engineering, Georgia Institute of Technology, Emory University, Atlanta, GA, United States; ^5^iHuman Institute, ShanghaiTech University, Shanghai, China

**Keywords:** resting-state fMRI, fMRI denoising, vascular modulation, head motion, arousal

## Introduction

Resting-state functional magnetic resonance imaging (rs-fMRI) has exponentially increased in adoption in the past decade. It is now a part of nearly every neuroimaging-based large-scale study of the human brain due to ease of use and versatility (Smith et al., 2013; Bookheimer et al., 2019; Zonneveld et al., 2019; Power, 2020). This has also led to the introduction of numerous derivative metrics. rs-fMRI is used in mapping the human-brain connectome and in understanding every aspect of cognitive function. It is also regarded as a promising biomarker for brain diseases ranging from dementia to traumatic brain injury to autism spectrum disorders. However, limitations on the sensitivity and specificity of rs-fMRI (Buckner et al., 2013) are a disadvantage. As we stand on the cusp of widespread adoption of rs-fMRI in clinical research, there is an urgent need to understand what we do not yet know about the origins of the rs-fMRI signal. This is the driving force behind our Research Topic, “Origins of the Resting-state fMRI Signal,” in which we strove to provide a comprehensive update on the quest for a better understanding.

## Neuronal Origins

The mechanism behind the rs-fMRI signal is the slight dissociation between the cerebral blood flow and oxygen consumption which gives the physiological basis to blood oxygenation level dependent (BOLD) signal (Kim and Ogawa, 2012). The hemodynamic BOLD signal fluctuations follow the ongoing neuronal activity changes through neurovascular coupling. While this process seems simple and straightforward it has limitations as Lu et al. describes in their mini-review, where they coin the term “neurocentric” model. They bring attention to that the spontaneous neuronal activities can explain only a small percentage of the variation of rs-fMRI fluctuations indicating that more complex neuropil activities together effect the spontaneous fluctuations. In this Research Topic two more original papers show the complex relationship between spontaneous neuronal activity and BOLD fluctuations. Zhang X. et al. describes the relationship between local field potentials (LFP) and simultaneously measured BOLD signal fluctuations in anesthetized rats. This relationship can be non-linear under isoflurane and linear under dexmedetomidine anesthesia, indicating that different brain states have real influence to the origin of rs-fMRI. Zhang Z. et al. demonstrates that the intrinsic functional connectivity depends on the neuronal activity patterns of different brain states. In monkey brains the isoflurane specific burst-suppression activity increased the functional connectivity compared to the stable slow wave activity of the same isoflurane anesthesia.

## Vascular Origins

While most studies use parameters derived from rs-fMRI as representing neuronal connectivity, it is important to realize that the BOLD rs-fMRI signal is not a direct measure of neuronal activity. Not only is the neuroanally-driven BOLD signal highly sensitive to vascular modulation (Liu, 2013), the frequency range of interest for rs-fMRI (i.e., below 0.1 Hz) is known to contain contributions from signals of non-neuronal origin. These factors have been of increasing interest in recent years (Liu, 2013; Golestani et al., 2016; Chu et al., 2018; Bright et al., 2020; Lewis et al., 2020), and are examined in this Research Topic. Tong et al., who were the first to demonstrate the unique vascular signature of low-frequency rs-fMRI fluctuations, present an update on the origins of these low-frequency vascular oscillations, their correction strategies and more interesting, ways in which they can be used in addressing clinical needs. Furthermore, Whittaker et al. investigate the relationship between systemic blood pressure and the rs-fMRI signal, while demonstrating the potential for the rs-fMRI signal to be used in tracking autoregulation.

## Cognitive Origins

If the rs-fMRI signal has a neural basis, its properties should be affected by widespread changes in activity linked to arousal. Two papers in this special issue address this question by examining rs-fMRI under different vigilance levels. Yin et al. compare awake humans to anesthetized monkeys and determine that functional flexibility is weakened but that its spatial distribution is somewhat preserved across species and states of consciousness. Liu and Falahpour review existing studies that link changes in vigilance to differences in BOLD signal amplitude and functional connectivity. Both papers indicate that similar time-varying patterns of activity are present in different vigilance states, but that the properties of the activity patterns vary with arousal.

## Noise and Artifactual Origins

As with all biological signals, some shared variance in rs-fMRI comes from noise. Subject motion creates noise, and Maknojia et al. provide a detailed review of its sources, prevention, and removal in post-processing. Physiological signals at a higher sampling rates than rs-fMRI (e.g., respiration and pulse) may alias, so Huotari et al. studied the effect of under-sampling on numerous rs-fMRI metrics. While dynamic rs-fMRI metrics were affected, rs-fMRI metrics calculated over 5 min remained stable. Yuen et al. separated rs-fMRI signals into intrinsic mode functions (IMFs) using variational mode decomposition (VMD). They suggest that some IMFs corresponded to physiological/metabolic processes combined with rs-fMRI networks, while other IMFs corresponded to vasomotor/unknown sources and did not correspond to rs-fMRI networks (Yuen et al.).  Moradi et al. applied empirical mode decomposition (EMD) to rs-fMRI and concluded that, vs. regressing the whole-brain “global” signal to remove noise, EMD provided IMFs with more spatially-specific measurements of global effects (Moradi et al.; Yuen et al.). Altogether, these studies suggest that the required sampling rates are dependent upon analysis method, and data-driven decomposition methods like VMD and EMD may provide signal decompositions that enhance noise removal.

## Perspectives On Future Work

Despite rs-fMRI's success as a research technique, we see challenges in the two primary application routes: (1) for neuroscience research, for which rs-fMRI has become a mainstay in human studies, but the difficulty of interpreting rs-fMRI metrics has limited application to basic science; (2) for clinical applications, where the lack of sensitivity and specificity has limited the adoption of rs-fMRI in assessing diseases. This Research Topic highlights the strong influence of arousal, vascular changes and noise contributions to rs-fMRI metrics, questioning the general practice of interpreting any rs-fMRI significance as being functional. For the foreseeable future, the community is urged to invest more effort into identifying the neuronal relevance of physiological and vascular oscillations in rs-fMRI, and into standardizing the definition of “resting state.”

## Author Contributions

All authors contributed equally to the writing of this editorial.

## Conflict of Interest

The authors declare that the research was conducted in the absence of any commercial or financial relationships that could be construed as a potential conflict of interest.
